# Football referee gesture recognition algorithm based on YOLOv8s

**DOI:** 10.3389/fncom.2024.1341234

**Published:** 2024-02-19

**Authors:** Zhiyuan Yang, Yuanyuan Shen, Yanfei Shen

**Affiliations:** School of Sport Engineering, Beijing Sport University, Beijing, China

**Keywords:** football gesture recognition, deep learning, YOLOv8s, GAM, P2 detection head, MDPIoU

## Abstract

Gesture serves as a crucial means of communication between individuals and between humans and machines. In football matches, referees communicate judgment information through gestures. Due to the diversity and complexity of referees’ gestures and interference factors, such as the players, spectators, and camera angles, automated football referee gesture recognition (FRGR) has become a challenging task. The existing methods based on visual sensors often cannot provide a satisfactory performance. To tackle FRGR problems, we develop a deep learning model based on YOLOv8s. Three improving and optimizing strategies are integrated to solve these problems. First, a Global Attention Mechanism (GAM) is employed to direct the model’s attention to the hand gestures and minimize the background interference. Second, a P2 detection head structure is integrated into the YOLOv8s model to enhance the accuracy of detecting smaller objects at a distance. Third, a new loss function based on the Minimum Point Distance Intersection over Union (MPDIoU) is used to effectively utilize anchor boxes with the same shape, but different sizes. Finally, experiments are executed on a dataset of six hand gestures among 1,200 images. The proposed method was compared with seven different existing models and 10 different optimization models. The proposed method achieves a precision rate of 89.3%, a recall rate of 88.9%, a mAP@0.5 rate of 89.9%, and a mAP@0.5:0.95 rate of 77.3%. These rates are approximately 1.4%, 2.0%, 1.1%, and 5.4% better than those of the newest YOLOv8s, respectively. The proposed method has right prospect in automated gesture recognition for football matches.

## 1 Introduction

Globally, football is considered the most popular sport, with a massive following of enthusiasts and spectators (Giulianotti, 2002; Wang et al., 2019; Li and Zhang, 2021; Bernardo et al., 2022). Referees play a crucial role in ensuring the smooth progression of football matches (Aragão e Pina et al., 2018). In addition to the main referee and assistant referees, the Video Assistant Referee (VAR; Spitz et al., 2021) also assists referees in making real-time decisions using video replays and communication technology to ensure the accuracy and fairness of rulings (Zglinski, 2022). However, this process often requires the VAR to focus on the screen and visually identify the referee’s decisions, which can be inefficient and impact the smooth flow of the game (Holder et al., 2022). Furthermore, as big data is increasingly applied to football, there has been a growing emphasis on the statistical analysis of referees’ decisions and performance evaluation. After the match, numerous football data websites promptly compile information about the type, quantity, and timing of the referee’s decisions for researchers to analyze. Professional sports teams also employ data analysts to analyze their opponents’ tactics and referees’ decisions during halftime breaks (Wright et al., 2013). Coaching staff can use these analytical insights to adjust their tactical strategies for the second half, aiming to enhance the team’s chances of winning (Pantzalis and Tjortjis, 2020). Currently, the collection of information about the type and quantity of referees’ decisions still relies on manual visual recognition, which is relatively inefficient. When collecting referee decision data within a short time frame, manual recognition can lead to omissions and errors. This poses a significant challenge for the staff. Moreover, the traditional recognition of referees’ gestures relies on manual visual identification, which requires a significant investment of manpower and resources to train the referees in this specialized skill. In addition, the evaluation of game results is subject to individual subjective judgments and biases, which have the potential to create disputes and compromise the overall fairness of matches (Boyko et al., 2007). Therefore, the development of a fast and accurate algorithm using computer technology for the automated recognition of football referees’ decisions is of paramount importance.

Depending on the type of sensor used, referee gesture recognition can be classified into two primary methods. One approach includes leveraging wearable devices to analyze data gathered from equipment worn by users. Despite the high accuracy of gesture recognition using wearable devices, these devices are expensive, require regular maintenance, consume significant resources, and are limited to non-competitive scenarios, thereby lacking real-time detection capabilities. The other type involves visual sensors that directly analyze the gestures within images and videos, which are further segmented into traditional and deep learning methods. Traditional vision-based methods typically rely on manually designed feature extractors and per-form poorly in complex scenes and multi-class gesture recognition. As deep learning continues to advance in the field of computer vision, many researchers have begun to use object detection algorithms to address the task of gesture recognition, such as the R-CNN (Girshick et al., 2014; Girshick, 2015; Ren et al., 2015) and YOLO (Redmon et al., 2016; Redmon and Farhadi, 2017; Redmon and Farhadi, 2018; Bochkovskiy et al., 2020; Li et al., 2022; Terven and Cordova-Esparza, 2023; Wang et al., 2023) series. Thanks to its exceptional precision and fast detection speeds, the YOLO series of algorithms is particularly well-suited for real-time target detection. The YOLO algorithm can effectively, accurately, and quickly recognize various hand gestures, and it benefits from mature deployment technology.

**Figure 1:**
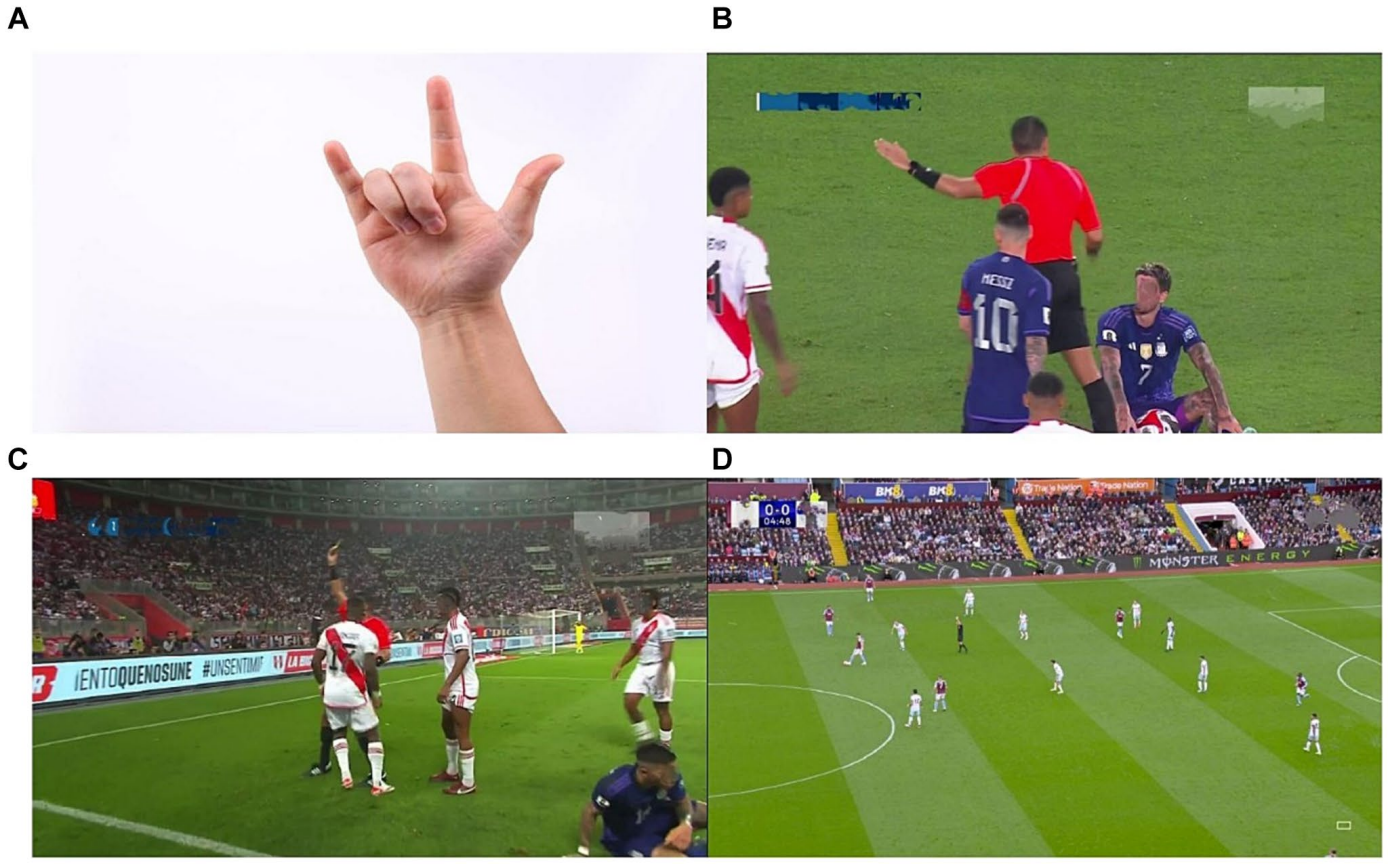
**(A)** Hand gestures with a relatively uniform background, angle, and scale; **(B–D)** Football referee gestures with complex backgrounds, obstructed visibility, blurring issues, and small targets.

However, it’s worth mentioning that the majority of previous studies were conducted under conditions that had a relatively consistent background, angle, and scale. In contrast, recognizing the hand gestures of soccer referees is significantly different from the previous studies, as shown in Figure 1.

Compared to generic gesture recognition task, the referee gesture recognition in our work involves a much more complex background. Football games are typically captured by multiple cameras, and the footage from each camera is usually managed by staff for the live broadcast. Our data is selected from live broadcast footage, thereby incorporating multiple angles and enhancing overall diversity. Typically, the scenes we capture include referees, athletes, and spectators all at once, introducing various sources of interference. Multiple cameras capturing images from different angles may result in the variable extraction of the same gesture features, as well as issues, such as occlusion and partial data loss. Furthermore, when long-distance cameras are used for recording, the interference factors become more pronounced, and the referees may appear smaller and blurrier, thereby increasing the complexity of gesture recognition. Additionally, the proper positioning of the referee is crucial to effectively observing the on-field situation and making appropriate judgments. Personnel also gather data on the referee’s positioning during decision making, which serves as an evaluation metric for the referee’s performance. Currently, most algorithms primarily annotate only the hands of individuals during data processing, making it challenging to accurately determine the person’s location.

To tackle the previously mentioned concerns, this research introduces the FRGR-YOLOv8s model, which is based on the YOLOv8s architecture. It aims to accurately identify the gestures made by football referees. This study provides the following five noteworthy contributions:

1. In this study, we created a dataset for soccer referee gestures. Unlike the typical approach of only annotating the hands, our study’s emphasis is on annotating the entire body of the referee, which enables improved recognition of the referee’s position.
2. In the context of identifying football referees’ gestures during a football match, challenges, such as background interference, occlusion, and partial visibility, frequently arise. The FRGR-YOLOv8s model incorporates a GAM (Liu et al., 2021) into the YOLOv8s model. It specifically focuses on the referees’ gestures, reducing the interference from background information, while also integrating the global context. This not only enhances the model’s performance, but also mitigates the risk of overfitting.
3. When using long-distance cameras for recording, the interference factors can become more noticeable, making it challenging to discern the referees who may appear to be small and blurry. In turn, this complicates the task of recognizing their gestures. To address this issue and improve the recognition of referees’ gestures captured using long-distance cameras, we have introduced a P2 detection head into the network, enhancing the detection of smaller objects.
4. The effective positioning of the referee is essential for accurately assessing on-field situations and making informed decisions. To enhance the referee’s position identification, we have improved the overall detection accuracy. Specifically, we have replaced the original CIoU (Zheng et al., 2020) with MPDIoU (Siliang and Yong, 2023), thereby more effectively leveraging the geometric properties of the referee gesture anchor boxes.
5. Through comparative and ablation experiments, our results demonstrate that FRGR-YOLOv8s outperforms the other baseline models in the task of recognizing football referees’ gestures.

## 2 Related work

### 2.1 Gesture recognition with wearable devices

In the field of wearable devices, information, such as the referee’s motion, posture, and the trajectory, is collected using wearable accelerometers, electromyography (EMG) signal sensors, inertial measurement units (IMU) sensors, and other devices. Subsequently, computer-based data processing and comparison are performed. Chambers et al. (2004) utilized the accelerometer data collected from wristbands and employed a hidden Markov model (HMM) along with various statistical feature sets to recognize the referees’ gestures in 10 types of cricket match. Yeh et al. (2017) employed EMG in combination with three-axis accelerometers to acquire motion signals. Pan et al. (2018) introduced a hybrid neural model called ORSNet, which utilizes wearable IMU signal sensors to identify 65 types of basketball referee signals, with an accuracy of 95.3%. Pan et al. (2020) used wearable sensors, including SEMG sensors and IMU, which was integrated with a device called an MYO arm band, to record the gestures made by referees. They utilized deep belief networks to generate training data for the recognition of violations and fouls in basketball referees’ gestures, com-paring them to the standard gestures. Despite the high accuracy of gesture recognition using wearable devices, these devices are expensive, require regular maintenance, consume significant resources, and are limited to non-competitive scenarios, thereby lacking real-time detection capabilities.

### 2.2 Gesture recognition with traditional computer vision

In terms of gesture recognition using traditional computer vision methods, Guyon et al. (2014) created the Chalearn gesture dataset using Kinect cameras, which includes wrestling and volleyball referees’ gestures. Žemgulys et al. (2018) introduced a method that relies on a histogram of oriented gradient (HOG) characteristics to categorize the individual basketball referees’ gestures within non-competitive video footage. They attained a 97.5% accuracy rate with the utilization of support vector machines (SVM). The data they used was downloaded from the internet. The referee stood in front of the camera, and all of his gestures were clearly visible. There are a total of 20 images featuring the referee, showcasing four different types of gestures. Subsequently, Žemgulys et al. (2020) subsequently enhanced an image segmentation method that leverages HOG and local binary patterns (LBP) characteristics. The data utilized was obtained from basketball game recordings downloaded from McDonald. In these recordings, the referee was positioned either facing the camera or with their back to the camera. In total, there are 100 images representing three different categories of gestures. This technique accurately identifies basketball referees’ gestures from basketball game recordings, achieving a 95.6% accuracy. Traditional methods typically rely on manually designed feature extractors and often perform poorly in complex scenes and multi-class gesture recognition.

### 2.3 Gesture recognition with deep learning

Given the advanced state of application technology for YOLO algorithms, they are now well-suited for mobile device deployment and adaptable in a wide range of scenarios (Zeng et al., 2023). Ni et al. (2018) presented a Light YOLO model designed for rapid gesture recognition in intricate environments. The data used was captured with individuals facing the camera, encompassing various lighting conditions and diverse background environments. Building upon YOLOv2, the researchers introduced spatial refinement modules and selective channel pruning methods. These improvements led to a precision increase from 96.80 to 98.06%, a growth from 40 FPS to 125 FPS, and a reduction in model size from 250 Mega Byte to 4 Mega Byte. Mujahid et al. (2021) introduced a compact model for gesture recognition that utilizes a combination of YOLOv3 and the DarkNet-53 convolutional neural network. The gesture data was captured from a frontal perspective, emphasizing the hands against a sample background. In total, there are 216 images covering 5 distinct gesture categories. This model attains a remarkable precision, even with challenging surroundings, and effectively identifies the gestures even when dealing with low-resolution images. Mesbahi et al. (2023) introduced an improved model that combines some elements from YOLOv4-tiny (Jiang et al., 2020) and YOLOv5. Their data was captured from a frontal perspective and includes commonly used sign language gestures. The objective of this model is to assist individuals who are deaf or have hearing impairments with video calls through the use of gestures. Wu et al. (2023) introduced an enhanced static gesture recognition algorithm built upon YOLOv5s. The gesture data they used was sourced from the Baidu PaddlePaddle Developer Forum, and it exclusively featured images of hands without the entire human body. There are a total of 14 common everyday gestures. This algorithm reduces the model’s parameters, while simultaneously enhancing its precision. It achieved an average precision increase of 3.1%. However, current gesture recognition algorithms are mainly designed to recognize gestures in uncomplicated backgrounds and at a single scale. In the context of soccer matches, where the backgrounds and environmental factors are complex and involve multiple cameras capturing gestures from different angles and distances, the model needs to be more adaptable to achieve effective recognition.

### 2.4 YOLOv8 model

**Figure 2:**
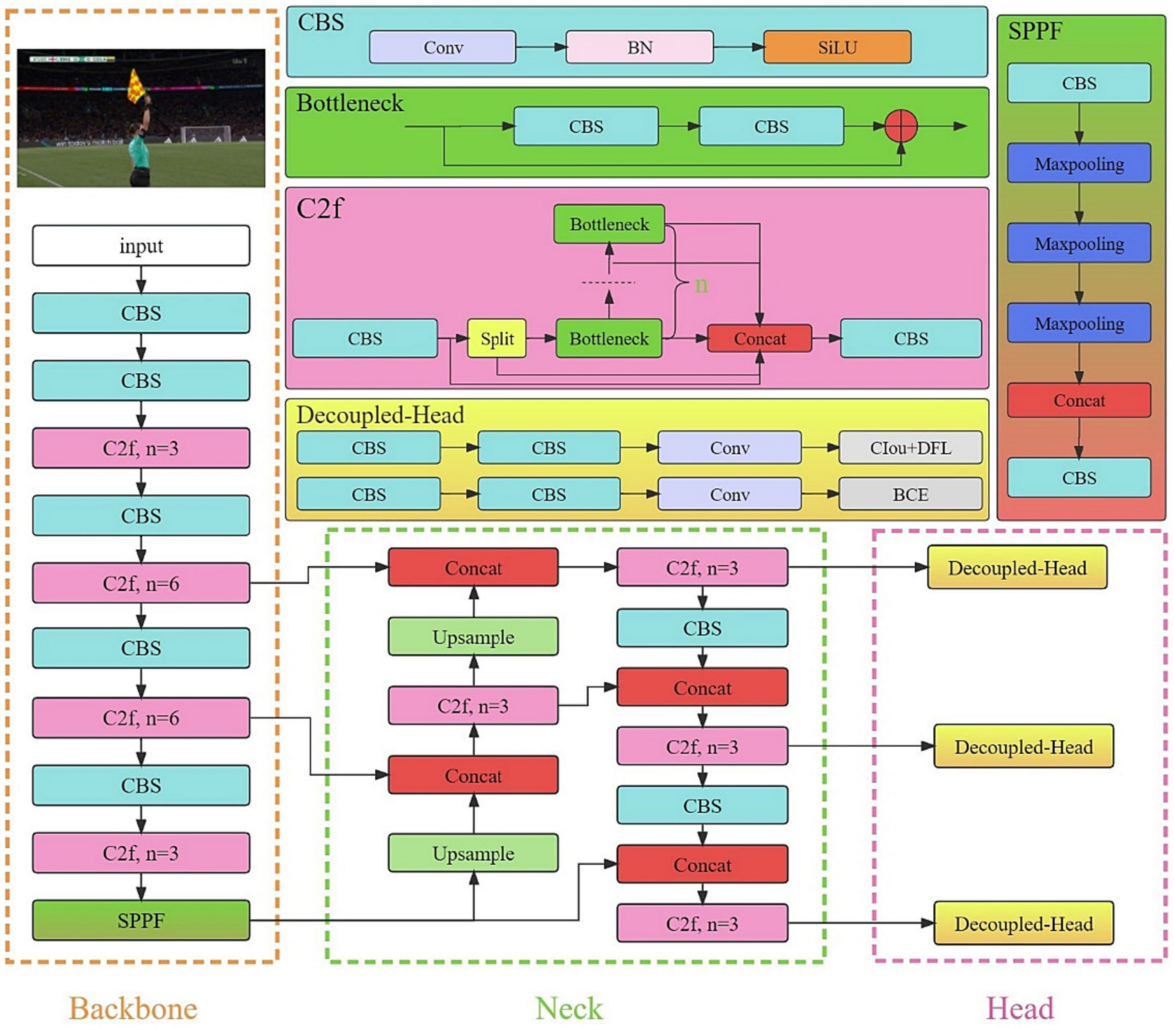
YOLOv8 model.

The YOLO models, which are all one-stage object detection algorithms, can simultaneously predict the categories and positions of multiple targets, offering a trade-off between speed and accuracy. Therefore, they are well-suited to solving gesture recognition problems in sports scenarios. YOLOv8, which was unveiled in 2023, expands upon the accomplishments of its predecessors by integrating novel attributes and enhancements aimed at boosting both performance and adaptability. The YOLOv8 model primarily consists of three network components: the backbone, neck, and head net-works, as shown in Figure 2.

The backbone network of YOLOv8 has been further optimized since the advent of YOLOv5. Firstly, the initial layer was modified from a 6 × 6 convolution to a 3 × 3 convolution. Additionally, the C2f (Sun et al., 2021) and C3 (Park H. et al., 2018) modules were optimized. It was de-signed with the intention of integrating E-ELAN (Gao et al., 2021). This enables the fusion of up-level features with the contextual data, improving the model’s detection accuracy, while maintaining network efficiency (Terven and Cordova-Esparza, 2023). Furthermore, the YOLOv8 network utilizes the SPPF (Jocher et al., 2022) module, which, based on the SPP (He et al., 2015) structure, successively guides the input through several layers of 5 × 5 max pooling. This effectively avoids the image distortion that may occur due to cropping and scaling images. Simultaneously, this method addresses the challenge of picking up repetitive features in convolutional neural networks, leading to significantly faster candidate box generation, while also reducing the computational costs.

The neck network in YOLOv8 continues to employ the PAN-FPN (Lin et al., 2017; Liu et al., 2018) architecture to create a feature pyramid for the YOLOv8 model, enabling the comprehensive integration of multi-scale information. The efficiency is enhanced by eliminating the upsampling convolutional stages from the PAN-FPN structure and directly feeding the features from various stages of the backbone network into the upsampling process. This modification contributes to an improved model efficiency.

The head network in YOLOv8 follows the prevalent Decoupled Head structure (Ge et al., 2021), which segregates the classification and detection components, leading to enhanced detection capabilities. The loss function can be conceptually separated into two key elements: classification loss, which integrates Binary Cross Entropy loss (BCE; Ruby and Yendapalli, 2020); and box loss, which encompasses both CIoU loss and distribution focal loss (DFL; Li et al., 2020).

## 3 The proposed methods

The YOLOv8 model has different configurations. This study improves upon the YOLOv8s model through the introduction of the FRGR-YOLOv8s model, which is specifically designed for football referee gesture recognition. The main enhancements incorporated into FRGR-YOLOv8s include:

1. We introduced a Global Attention Module (GAM) during the feature extraction phase to make the model focus more on the referees’ gestures, reducing the interference from complex backgrounds.
2. We incorporated a P2 small object detection head to enhance the recognition performance of the referees’ gestures captured at a distance, especially those that are small and blurry.
3. We used MPDIoU instead of CIoU. This resulted in an increased model accuracy and faster convergence speed. This allows the better prediction of the referee gestures’ positions, enhancing the detection accuracy.

**Figure 3:**
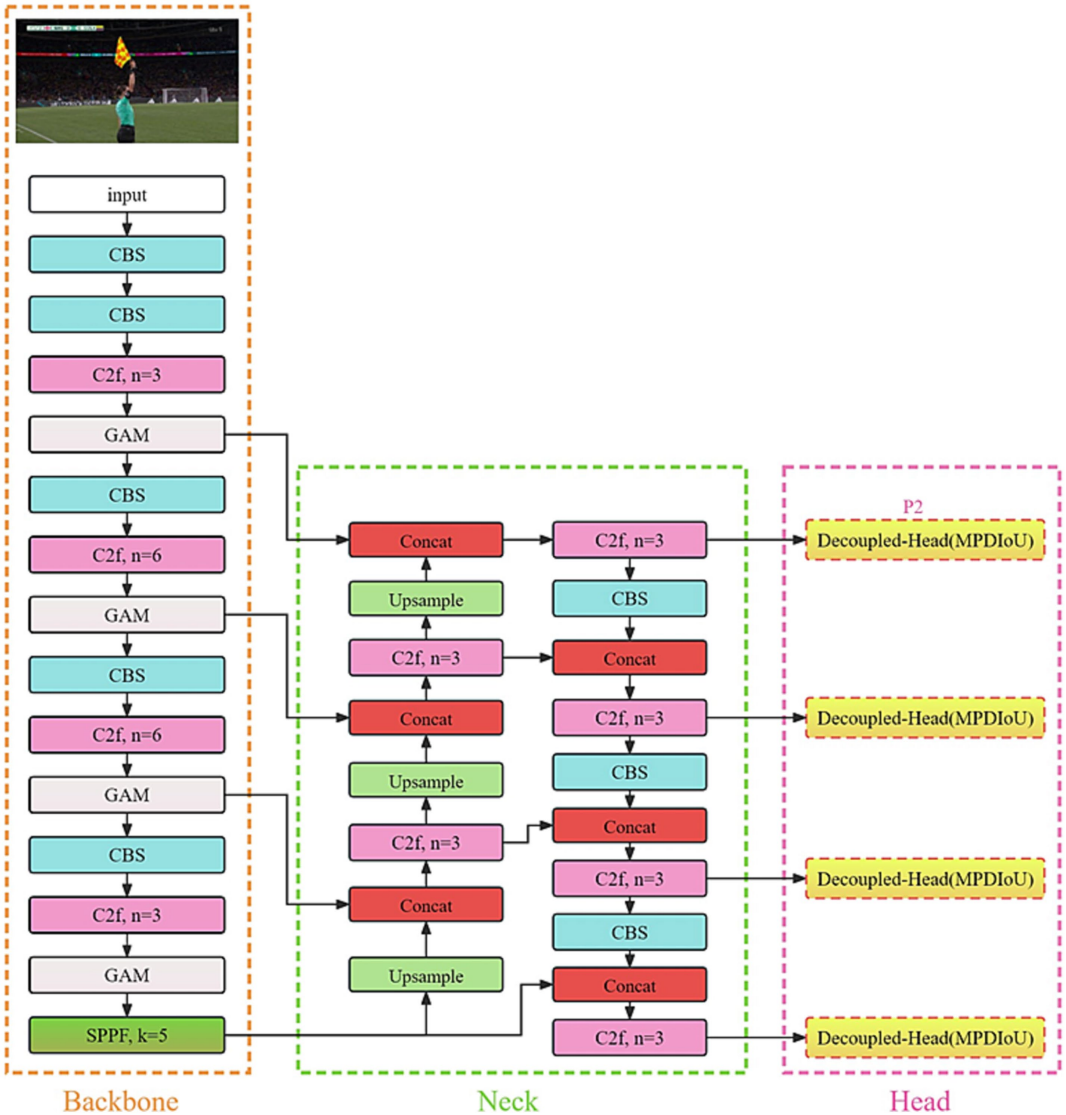
FRGR-YOLOv8s model.

The enhanced FRGR-YOLOv8s model is depicted in Figure 3.

### 3.1 Global attention module

The images in the dataset include the football referees and various background elements. During football referee gesture recognition, we focused on the referee’s arms. The attention mechanisms are heuristic methods in deep learning that operate similarly to human visual and cognitive systems. These mechanisms enable neural net-works to focus on relevant information during the processing of input data, ultimately enhancing the performance and generalization abilities of the model. YOLOv8s loses some crucial information related to the referees’ gestures during downsampling feature extraction in the main network. Integrating an attention mechanism into the feature extraction stage of the YOLOv8s model improves the extraction of features related to the referee’s arm while reducing background interference.

SE (Hu et al., 2018) introduced channel attention and feature fusion for the first time, but its efficiency is comparatively low, and it does not integrate spatial information. CBAM (Woo et al., 2018) and BAM (Park J. et al., 2018) improved performance by integrating channel attention and spatial attention modules. However, neither of these approaches fully considered the mutual relationship between channels and spatial dimensions, leading to the loss of cross-dimensional information. GAM, which was proposed by Liu and his colleagues, is a type of attention mechanism. The GAM model comprehensively incorporates spatial and channel attention mechanisms, emphasizing the interaction between spatial and channel dimensions. This comprehensive approach helps to capture feature correlations more effectively, thereby enhancing model performance. By simultaneously emphasizing spatial and channel attention, the GAM effectively addresses the shortcomings of the previously mentioned models in handling cross-dimensional information.

**Figure 4:**
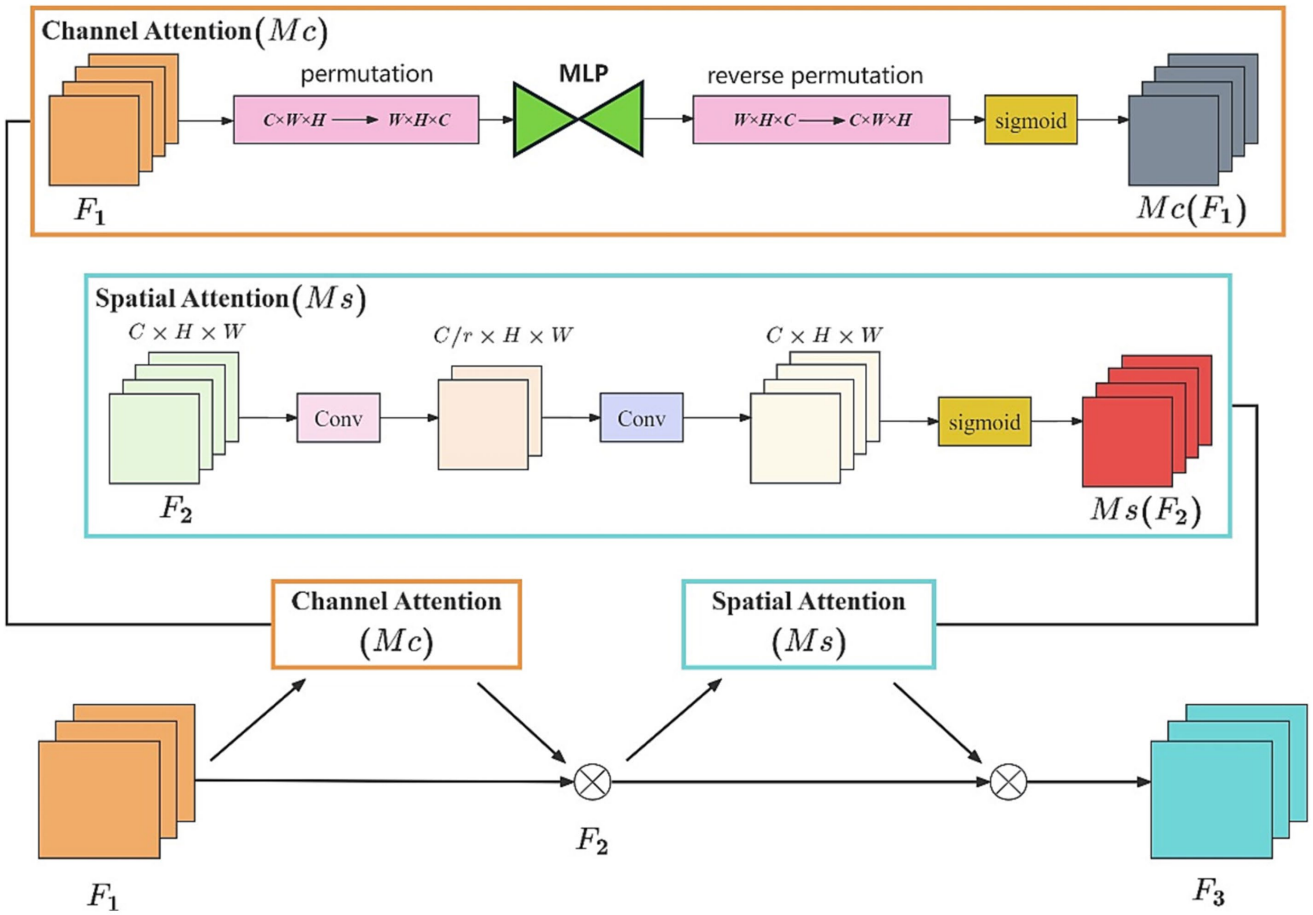
The GAM module.

GAM consists of both channel attention (Mc) and spatial attention (Ms) modules, as shown in Figure 4. The Mc is used to enhance the importance of specific channels to capture more task-related information. Meanwhile, the Ms. is employed to focus on specific regions of an image, facilitating a better understanding of the relationships between different areas in the image. Therefore, GAM is capable of integrating channel and spatial information, while focusing on capturing the key aspects of the referees’ gestures.

Given the input $F_{1}$, the intermediate variable $F_{2}$ and the result $F_{3}$ can be shown as follows:


$$F_{2}=F_{1}⊗Mc\left(F_{1}\right)$$

$$F_{3}=F_{2}⊗Ms\left(F_{2}\right)$$

Here, Mc represents channel attention, and Ms. represents spatial attention, respectively, with ⊗ indicating a component-wise multiplication.

### 3.2 P2 detection head

When images are taken with a long-distance camera, the gestures of the referee may become small and blurry. In the YOLOv8s model, the main network conducts downsampling through convolution during feature extraction to expand the receptive field. Subsequently, it undergoes multi-scale feature fusion via PAN-FPN.

FPN is structured by downsampling high-resolution feature maps and upsampling low-resolution feature maps, connecting them to create a pyramid configuration. During this process, the information from each layer of the feature maps is fused with that of the neighboring layers, allowing the target information to be preserved in higher-level feature maps; the background information from lower-level feature maps complements this. This processing enhances the model’s accuracy in multi-scale detection tasks.

PAN is designed to merge feature maps from different levels, ensuring efficient utilization of information within each feature map to enhance detection accuracy. PAN, much like FPN, uses a pyramid-style feature extraction network, but employs a bottom-up feature propagation technique. PAN’s design commences with upscaling from low-resolution feature maps and downscaling from high-resolution feature maps, merging them to create a single pathway. Throughout this process, the information from each layer of feature maps combines with that from the neighboring layers. However, in contrast to FPN, PAN sequentially concatenates the outcomes of the merged feature maps at different levels, instead of summing them. This sequential approach avoids information loss during summation and retains the finer details, thereby enhancing the detection accuracy.

**Figure 5:**
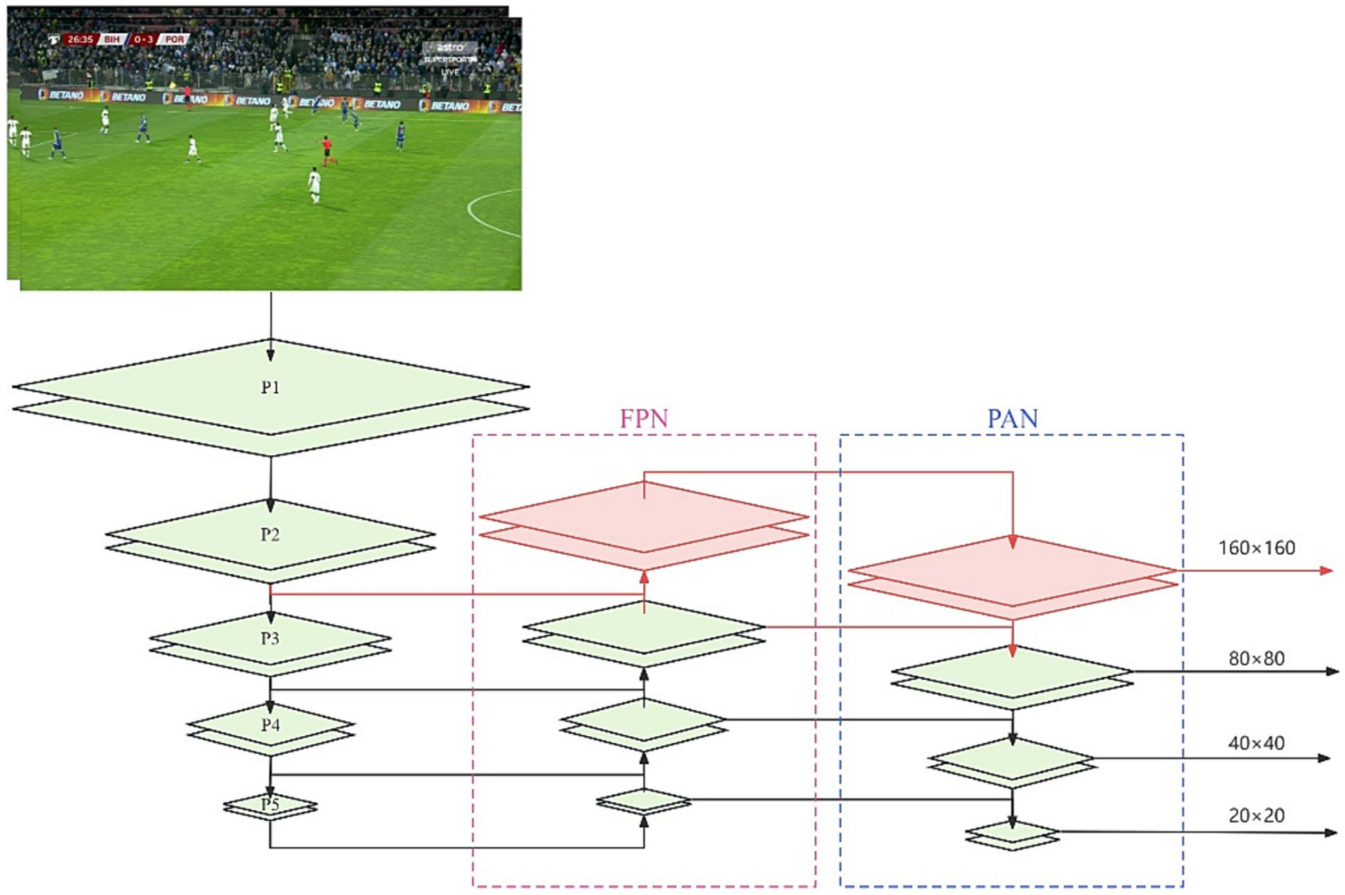
The red section represents the newly added P2 detection head.

Despite the multi-scale feature fusion in YOLOv8s, as the receptive field increases, the information about the small objects gradually diminishes. When the input image is set to 640 × 640, we obtain three different-sized feature map detection heads, namely 80 × 80(P3), 40 × 40(P4), and 20 × 20(P5). The P3 detection head shows superior performance in detecting medium-sized objects, while the P4 detection head excels in detecting large objects. The P5 detection head is the most effective for detecting extra-large objects. Given that most general datasets primarily consist of large and medium-sized objects, our soccer referee gesture dataset notably contains a significant number of small objects. To better detect the small objects, we introduced a P2 detection head with a feature map size of 160 × 160, as shown in Figure 5. The P2 detection head, with its larger feature maps, can incorporate more information about the small objects, thereby enhancing the recognition of the referees’ gestures in long-distance camera shots.

### 3.3 MPDIoU replaces CIoU

In official football matches, multiple cameras are placed at different distances, simultaneously recording the game. The same referee gesture may appear in different frames at various angles and distances, but the size relationship of the bounding boxes for the same gesture remains generally consistent across different frames. To enhance the FRGR-YOLOv8s model’s ability to effectively recognize football referees’ gestures, we should leverage this characteristic. Traditional CIoU loss can be ineffective when the predicted bounding box for a referee’s gesture has a similar aspect ratio to the ground truth box, but has a significantly different width and height. MPDIoU loss leverages the geometric properties of the football referees’ gesture bounding boxes to address this issue.

Intersection over Union (IoU) is a metric utilized to quantify the overlap between the predicted and ground truth bounding boxes. When IoU surpasses a specific threshold, it is typically indicative of successful target detection by the model. This threshold can be adjusted according to the specific requirements of the task. The calculation formula for IoU is as follows:


$$IoU=\frac{\left|B_{gt}\cap B_{prd}\right|}{\left|B_{gt}\cap B_{prd}\right|}$$

In this equation, $B_{gt}$ denotes the ground truth bounding box area, and $B_{prd}$ denotes the predicted bounding box area. IoU is also limited in certain situations. For example, when the value of $B_{gt}\cap B_{prd}$ is 0, it cannot provide a precise indication of the proximity between $B_{gt}$ and $B_{prd}$. To overcome some of these IoU limitations, other researchers and engineers have proposed many alternative evaluation metrics and improvement methods, such as GIoU (Rezatofighi et al., 2019), CIoU, DIoU, SIoU (Gevorgyan, 2022), EIoU (Zhang et al., 2022), MPDIoU, etc. They aimed to address some of the issues with IoU and improve the object detection performance.

**Figure 6:**
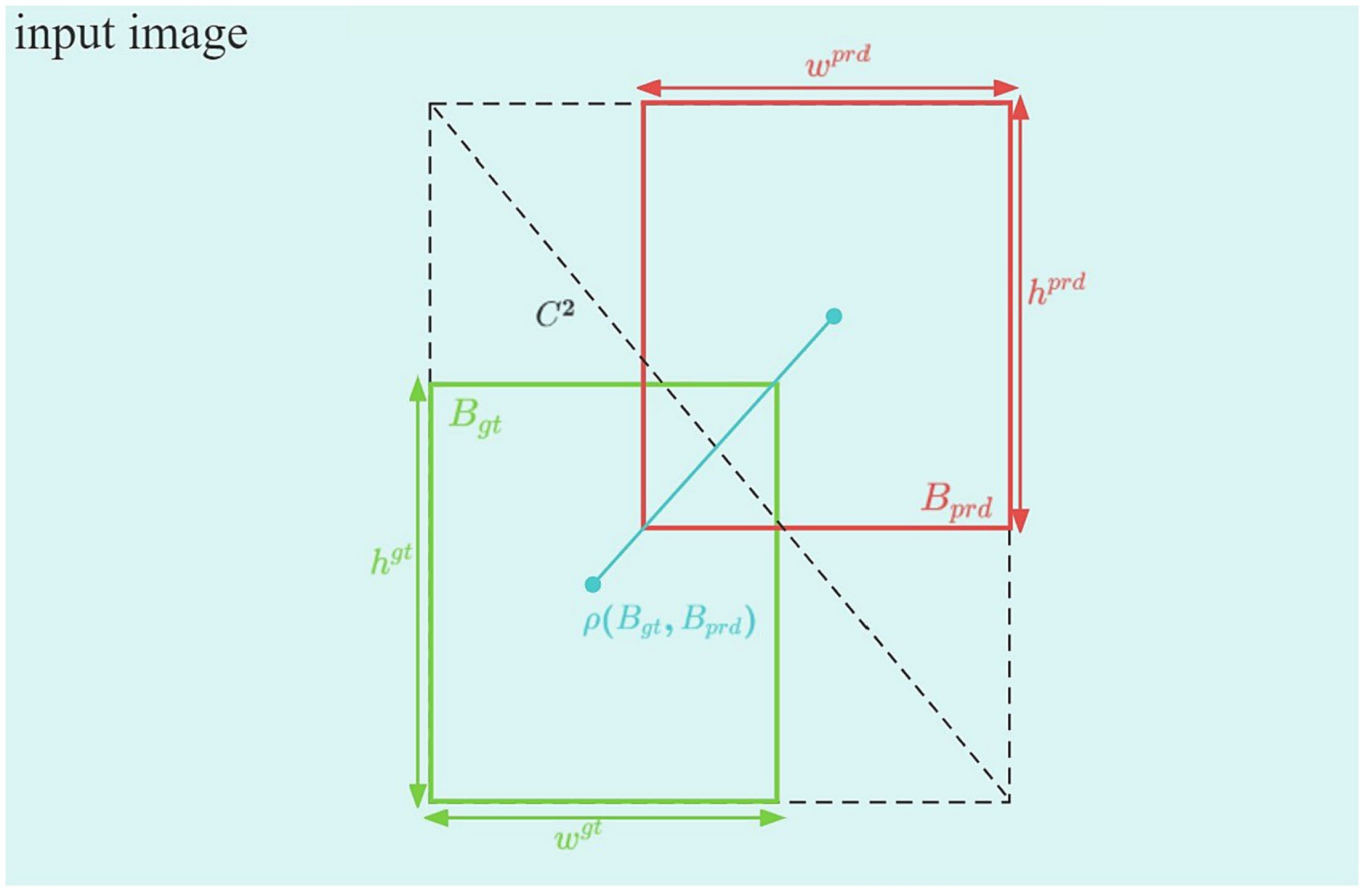
CIoU diagram.

CIoU is used in YOLOv8s, as shown in Figure 6.

CIoU can be expressed in the following manner:


$$\mathrm{CIoU}=IoU-\frac{\rho^{2}\left(B_{gt},B_{prd}\right)}{C^{2}}-\alpha V$$

where the expressions of V and α, respectively, are


$$V=\frac{4}{\pi^{2}}{\left(\arctan\frac{w^{gt}}{h^{gt}}-\arctan\frac{w^{prd}}{h^{prd}}\right)}^{2}$$

$$\alpha =\frac{V}{1-IoU+V}$$

Here, $C^{2}$ denotes the diagonal length of the smallest box covering $B_{gt}$ and $B_{prd}$. $\rho^{2}\left(B_{gt},B_{prd}\right)$ represents the Euclidean distance between $B_{gt}$ and $B_{prd}$, respectively. $w^{gt}$ and $w^{prd}$ represent the widths of the predicted and real boxes, respectively. Similarly, $h^{gt}$and $h^{prd}$ represent the matching degree between $B_{gt}$ and $B_{prd}$, respectively. This matching degree is more accurately determined by considering other factors, such as the intersection, union, center point distance, and the differences in width and height.

**Figure 7:**
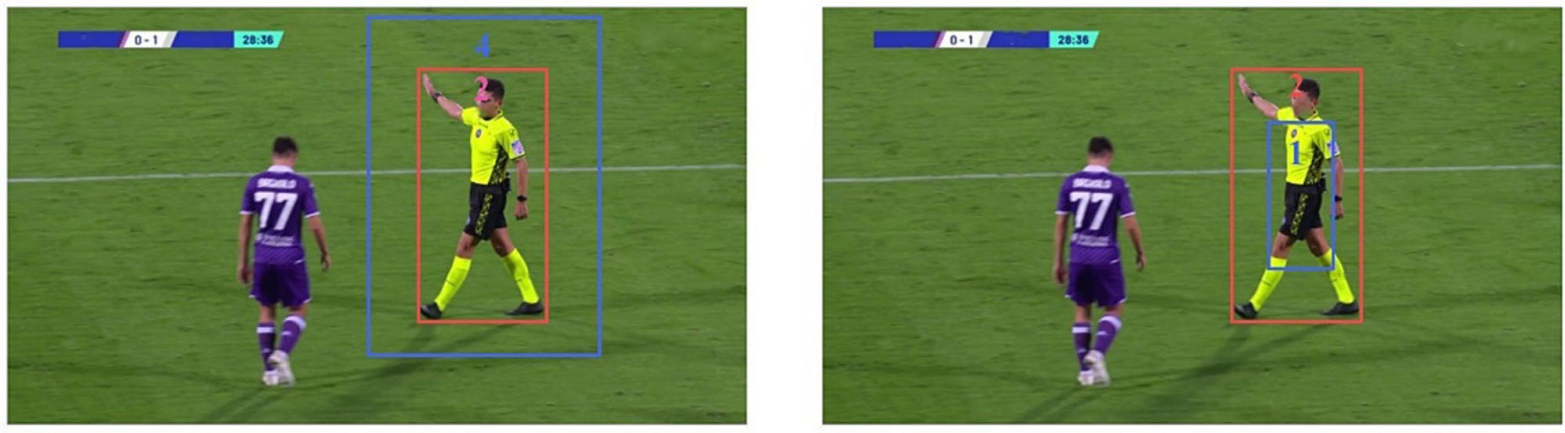
In two different scenarios, the real box remains the same, while the predicted box differs. However, the CIoU result remains consistent. Red indicates the real box, while blue indicates the predicted box.

The use of CIOU in YOLOv8s as an evaluation index for object detection models helps to enhance the efficiency of the models, particularly when dealing with objects that have different shapes, sizes, and positions. However, CIOU cannot detect when $B_{gt}$ has the same aspect ratio as $B_{prd}$, but its width and height are completely different. This constraint adversely affects both the model’s accuracy and convergence speed, as shown in Figure 7.

**Figure 8:**
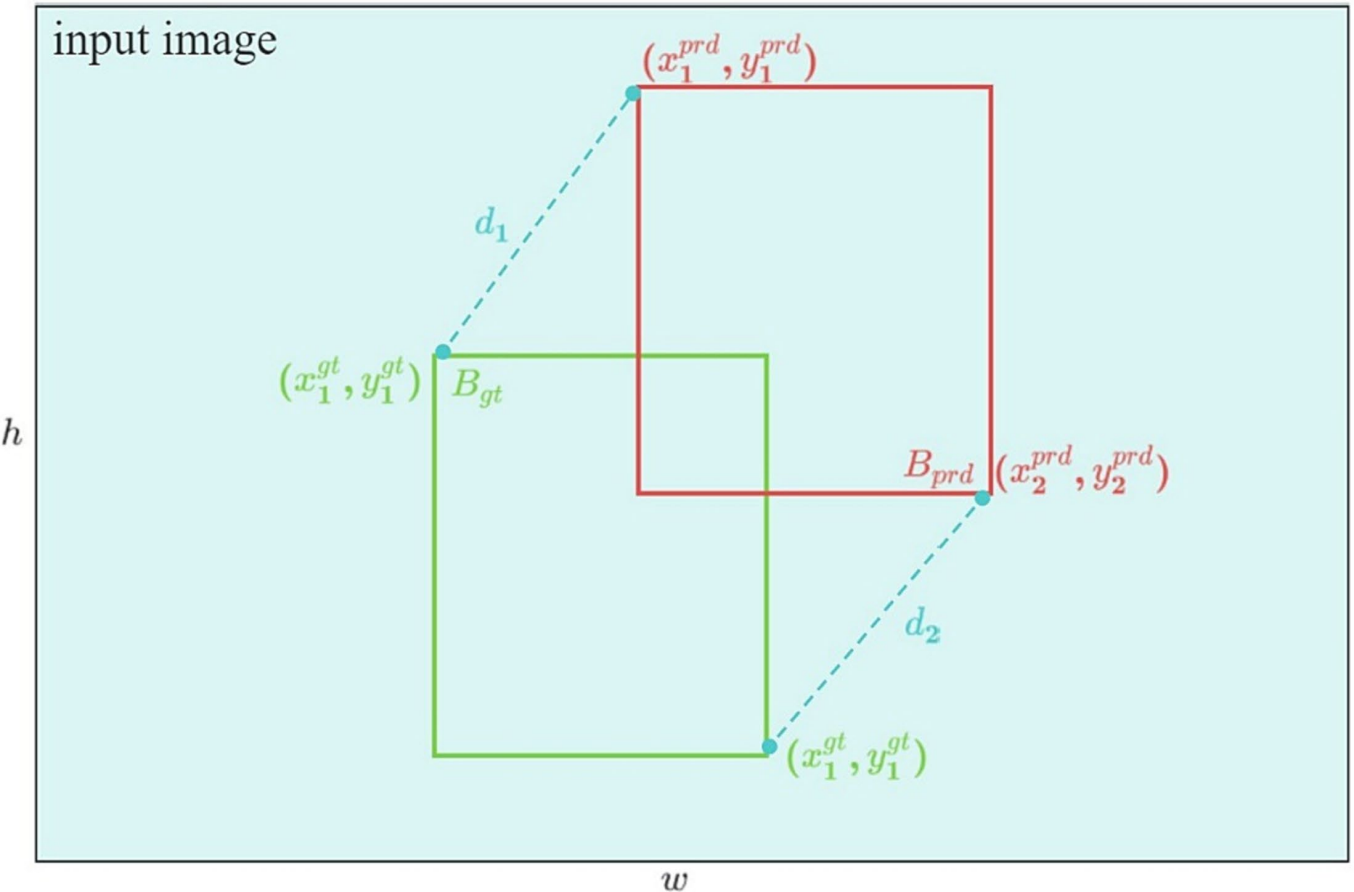
MPDIoU diagram.

To address the issues mentioned earlier, Ma et al. introduced MPDIoU loss. By harnessing the geometric characteristics of bounding box regression, MPDIoU loss can proficiently train the model to minimize the distance between the $B_{prd}$’s upper left and lower right corners and $B_{gt}$. This is achieved even when the aspect ratio of the referee gesture image remains consistent, but the length differs. MPDIoU is shown in Figure 8.

With the coordinates $\left(x_{1}^{prd},y_{1}^{prd},x_{2}^{prd},y_{2}^{prd}\right)$ and $\left(x_{1}^{gt},y_{1}^{gt},x_{2}^{gt},y_{2}^{gt}\right)$ of the upper left and lower right corners of $B_{prd}$ and $B_{gt}$, the distance $d_{1}$ between the upper left corners of $B_{gt}$ and $B_{prd}$ can be expressed as follows:


$$d_{1}^{2}={\left(x_{1}^{prd}-x_{1}^{gt}\right)}^{2}+{\left(y_{1}^{prd}-y_{1}^{gt}\right)}^{2}$$

and the distance $d_{2}$ between the lower right corners of $B_{gt}$ and $B_{prd}$ can be written as


$$d_{2}^{2}={\left(x_{2}^{prd}-x_{2}^{gt}\right)}^{2}+{\left(y_{2}^{prd}-y_{2}^{gt}\right)}^{2}$$

The expression formula of MPDIoU is


$$\mathrm{MPDIoU}=IoU-\frac{d_{1}^{2}}{h^{2}+w^{2}}-\frac{d_{2}^{2}}{h^{2}+w^{2}}$$

MPDIoU loss can be written as


$$L_{\mathrm{MPDIoU}}=1-\mathrm{MPDIoU}$$

## 4 Experiments and results

### 4.1 Dataset

The quality of a dataset significantly impacts the design and training results of object detection algorithms. Currently, there is a lack of openly accessible datasets of football referee gestures intended for deep learning applications. Therefore, this study utilized a self-created dataset of football referees’ gestures.

We compile our dataset through the following three steps: (1). Collecting football match videos from online sources and extracting images of referee gestures from the videos. (2). Annotating the images. (3). Partitioning the dataset into a test set and a training set. The specific process for establishing the dataset is outlined below.

**Figure 9:**
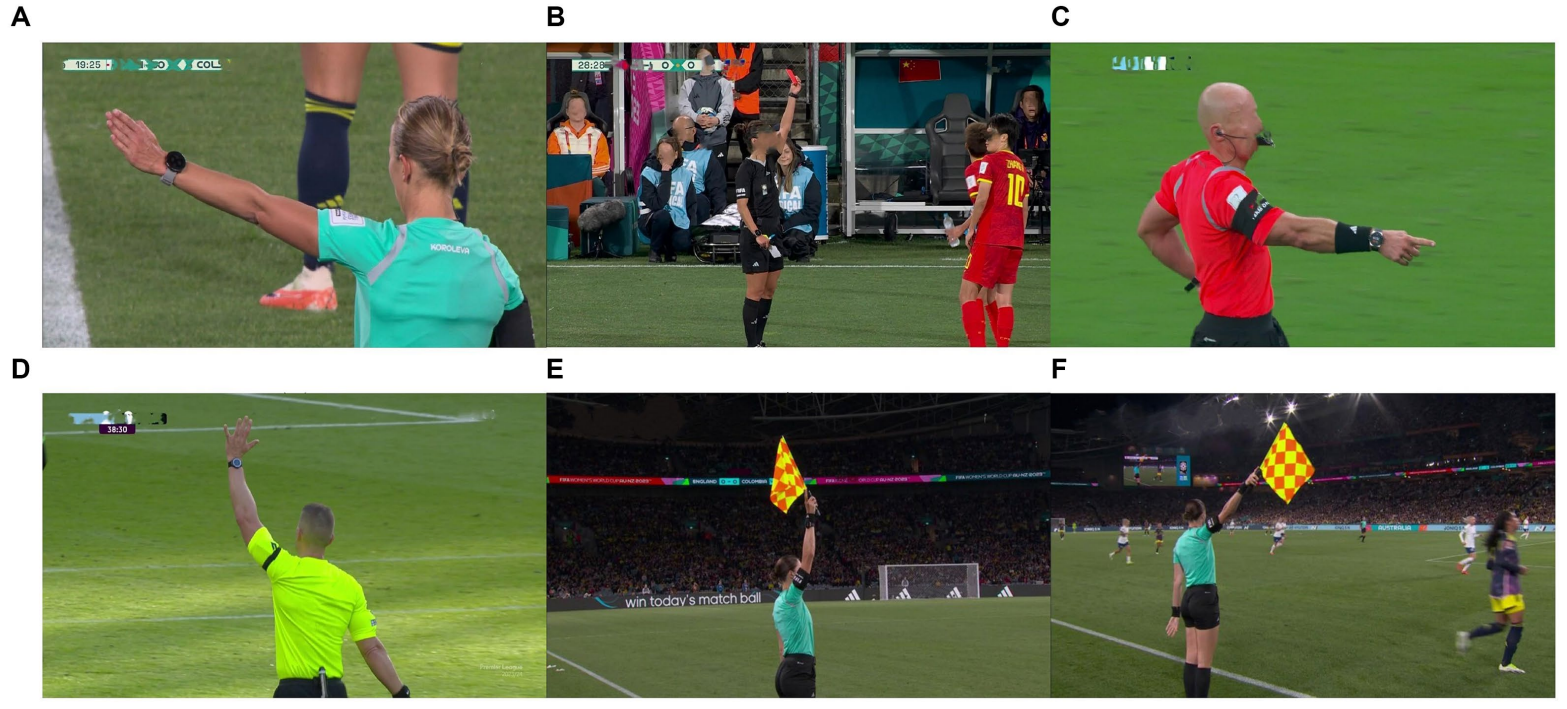
The dataset contains six types of football referee gestures. **(A)** The direct free kick gesture of the main referee. **(B)** Main referee’s cards gesture. **(C)** Main referee’s penalty kick gesture. **(D)** Main referee’s indirect free kick gesture. **(E)** Assistant referee’s offside gestures. **(F)** Assistant referee’s direct free kick gesture.

Our dataset is derived from video recordings of matches from the World Cup and the top five European football leagues spanning from 2018 to 2023. Considering that a referee’s gesture may persist across consecutive frames in original match videos, we treat these gestures as static and include only one frame as a representative sample in our dataset. Recognizing the importance of referee gestures in matches, our emphasis is on analyzing six common gestures that significantly influence match outcomes, as shown in Figure 9. In our experiments, we use Kinovea to carefully select referee pose frames that meet the requirements from the original videos and save them as image samples. Ultimately, we have successfully assembled a dataset comprising 1,200 images of referee gestures. In different matches and stadiums, the number and placement of cameras often vary. Moreover, there are differences in environmental lighting conditions and the colors of referee attire. As a result, the distinctive feature of this dataset is its ability to capture the diversity present in various match environments, including alterations *in camera* layouts, variations in lighting conditions, and differences in referee attire colors. This diversity plays a crucial role in strengthening the model’s robustness, allowing it to adapt more effectively to changes in different match scenarios.

**Figure 10:**
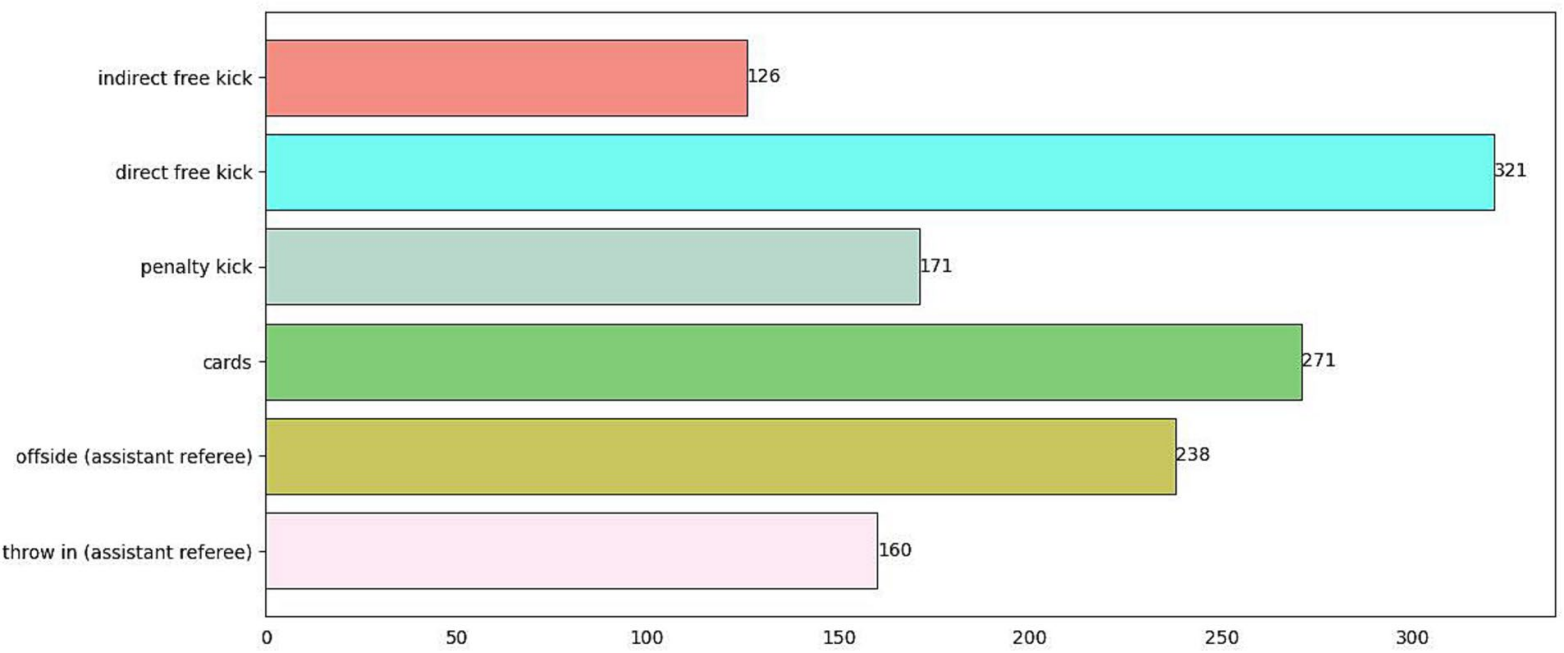
The quantity of each class sample. The horizontal axis represents the quantity of samples, while the vertical axis represents the category names for gesture recognition.

For the entire dataset we collected, each sample was annotated using LABELIMG in two aspects: (1) the referee’s positional information; (2) the category information of the referee’s gestures. Considering that the referee’s position is a crucial factor for further analyzing referee performance, we annotated the entire referee region rather than just the referee’s hand gestures. The final annotation information includes four numerical values: the x-coordinate and y-coordinate of the center of the annotation box, as well as the width and height of the annotation box. For the referee’s gesture type, we annotated it as one of the six categories shown in Figure 9. Additionally, the quantity of each class sample is depicted in Figure 10.

To ensure the accuracy and generalization ability of the model, we divide the whole dataset into training and testing sets, randomly splitting them in a ratio of 8:2.

### 4.2 Evaluation indicators

To assess the algorithm’s performance, we opted for precision (P), recall (R), and mean average precision (mAP) as the evaluation criteria for the detection performance.

P evaluates the correctness of positive predictions generated via the algorithm. R assesses the algorithm’s capacity to detect all the pertinent instances. P and R are defined as follows:


$$P=\frac{TP}{TP+FP}$$

$$R=\frac{TP}{TP+FN}$$

where TP corresponds to the true positives, FP corresponds to the false positives, and FN corresponds to the false negatives.

AP stands for the area under the P-R curve, while mAP is the average of the APs across various categories. The mAP evaluates the precision at different recall levels, and then computes the average precision across those levels. A higher mAP indicates a better performance in object detection tasks. AP and mAP are represented as follows:


$$AP=\overset{1}{\underset{0}{\int }}P\left(R\right)dR$$

$$\mathrm{mAP}=\frac{\sum_{1}^{K}AP}{K}$$

In this study, there are six categories of football referee gestures, so K = 6.

### 4.3 Experimental environment and training parameters

All the experiments in this study used the same machine, as indicated in Table 1.

**Table 1:** Experimental environment.

Configuration	Environment
CPU	Intel(R) Xeon(R) Platinum 8255C CPU @ 2.50GHz
GPU	RTX 2080 Ti
Operating system	Ubuntu 18.04.5
Video memory	11GB
Deep learning framework	Pytorch
Accelerated environment	CUDA 11.1

The models in this experiment were all trained with identical training parameters, as shown in Table 2.

**Table 2:** Training parameters.

Parameter	Value
Epochs	300
Batch	16
Image size	640 × 640
Workers	8
Learning rate	0.01

### 4.4 Experiments and results

#### 4.4.1 Comparison experiment

**Figure 11:**
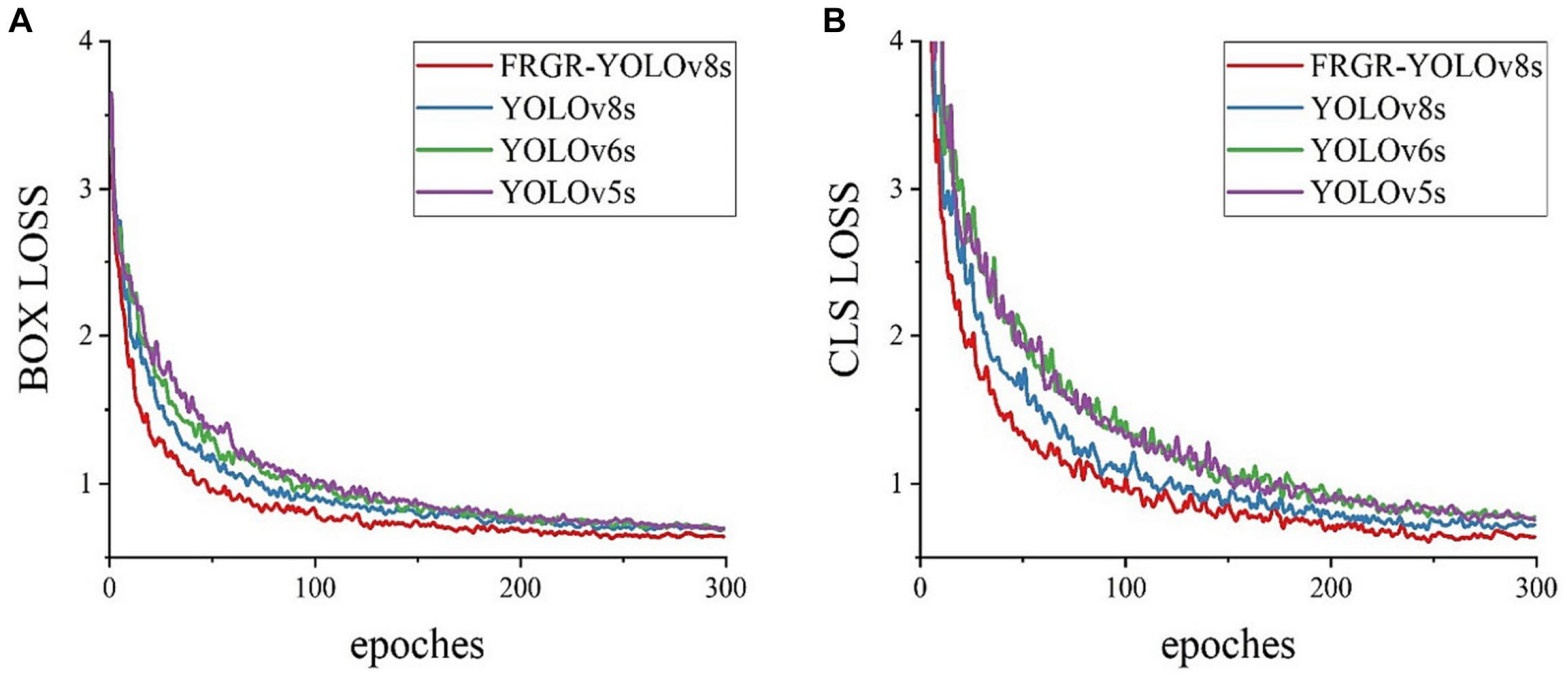
**(A)** Box loss; **(B)** Classification loss (CLS).

In this study, the comparison of training losses was conducted between our pro-posed FRGR-YOLOv8s model and the other models, including YOLOv8s, YOLOv5s from Ultralytics, and YOLOv6s from Meituan, as shown in Figure 11. Figure 10 high-lights several key observations. The FRGR-YOLOv8s model achieved the lowest box loss and converged the fastest. This is because the FRGR-YOLOv8s model introduced MPDIoU loss, which enabled the model to effectively utilize the geometric properties of the detection boxes. The FRGR-YOLOv8s model achieved the lowest classification loss and converged the fastest. This is because the inclusion of the GAM module led to a significant enhancement in the classification loss of the FRGR-YOLOv8s model. The GAM module significantly improved the model’s feature extraction capabilities across various gesture classes, while reducing the interference from shared features among different gesture classes. Overall, the FRGR-YOLOv8s model demonstrated an excel-lent performance in terms of training losses and outperformed the YOLOv8s, YOLOv6s, and YOLOv5s models.

**Figure 12:**
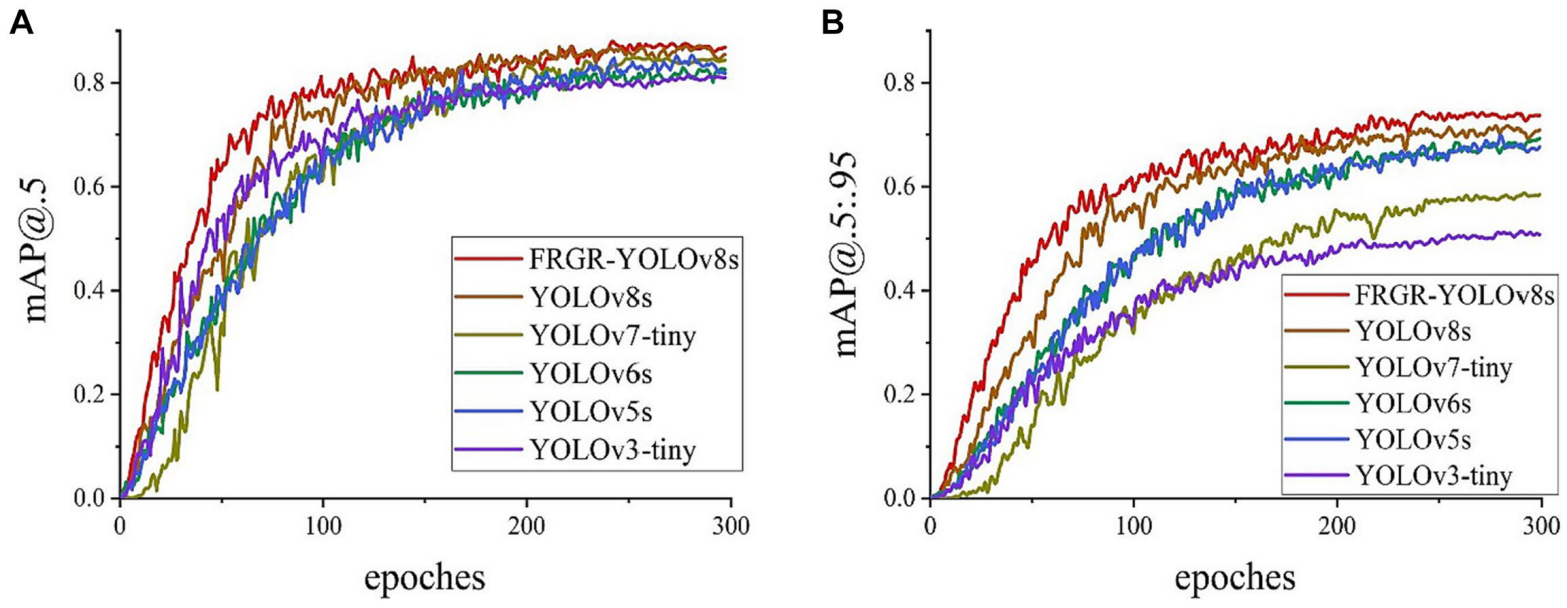
**(A)** The mAP@0.5 change curves of the 6 models; **(B)** The mAP@0.5:0.95 change curves of the 6 models.

In addition, comparative experiments were also carried out to assess the variations in mAP@0.5 and mAP@0.5:0.95 during the FRGR-YOLOv8s model training process and those of the YOLOv8s, YOLOv7tiny, YOLOv6s, YOLOv5s, and YOLOv3tiny models using the referee gesture dataset, as shown in Figure 12. The curves of various colors represent distinct models. The FRGR-YOLOv8s model consistently maintains an advantage in terms of mAP@0.5 and mAP@0.5:0.95 throughout the entire training process compared to the other models. This suggests that the FRGR-YOLOv8s model exhibits improved target detection capabilities in referee gesture recognition, resulting in a higher accuracy. Furthermore, the FRGR-YOLOv8s model not only demonstrates a superior performance, but also converges faster during training. So, the FRGR-YOLOv8s model can attain a superior performance within a reduced training period, which is a critical factor in real-world applications. In the context of the mAP scores, the FRGR-YOLOv8s model performs better than the YOLOv8s, YOLOv7tiny, YOLOv6s, YOLOv5s, and YOLOv3tiny models do.

In addition, we conducted a comparative analysis of the YOLOv8s, YOLOv7tiny, YOLOv6s, YOLOv5s, and YOLOv3tiny models on the test set. We compared P, R, mAP@0.5, and mAP@0.5:0.95, as shown in Table 3.

**Table 3:** Comparison of results for 6 models.

Models	*P*	R	mAP@0.5	mAP@0.5:0.95
Faster R-CNN	78.3%	76.8%	78.5%	59.7%
SDD	75.1%	74.6%	76.7%	56.9%
Yolov3tiny	83.1%	85.0%	86.6%	56.6%
Yolov5s	84.0%	85.6%	86.4%	70.4%
Yolov6s	85.1%	85.7%	87.8%	71.2%
Yolov7tiny	86.7%	86.4%	85.1%	59.2%
YOLOv8s	87.9%	87.9%	88.8%	71.9%
FRGR-YOLOv8s	89.3%	88.9%	89.9%	77.3%

The FRGR-YOLOv8s model achieved a P of 89.3%, which represents an improvement of 1.4, 2.6, 4.2, 5.3, 6.2, 12.2 and 10.0% over the YOLOv8s, YOLOv7tiny, YOLOv6s, YOLOv5s, YOLOv3tiny, SDD and Faster R-CNN models, respectively. The FRGR-YOLOv8s model achieved a recall of 88.9%, which represents an improvement of 1.0, 2.5, 3.2, 3.3, 3.9, 14.3 and 12.1% over the YOLOv8s, YOLOv7tiny, YOLOv6s, YOLOv5s, YOLOv3tiny, SDD and Faster R-CNN models, respectively. The FRGR-YOLOv8s model achieved an mAP@0.5 of 89.9%, which is an improvement of 1.1, 4.8, 2.1, 3.5, 3.3, 13.2 and 11.4% over the YOLOv8s, YOLOv7tiny, YOLOv6s, YOLOv5s, YOLOv3tiny, SDD and Faster R-CNN models, respectively. The FRGR-YOLOv8s model achieved a mAP@0.5:0.95 of 77.3%, which represents an improvement of 5.4, 18.1, 6.1, 6.9, 20.7, 20.4 and 17.6% over the YOLOv8s, YOLOv7tiny, YOLOv6s, YOLOv5s, YOLOv3tiny, SDD and Faster R-CNN models, respectively. In summary, the FRGR-YOLOv8s model exhibits a superior detection performance using the referee gesture dataset.

#### 4.4.2 Grad-CAM comparison

Grad-CAM (Zhou et al., 2016) calculates the corresponding weights via backpropagation of the class confidence score gradients and generates Grad-CAM visualizations. These Grad-CAM visualizations effectively highlight the key features in the referees’ gesture images, enabling us to better identify the highly repetitive details within the images and obtain more comprehensive texture information. In these Grad-CAM visualizations, every pixel represents the target or confidence score in its corresponding image position. When lower-level features are extracted accurately, the scores for the textures and other details tend to be higher. In turn, this results in brighter and more prominent regions in the Grad-CAM visualizations. These highlighted areas correspond to the important image regions that the model focuses on, thereby increasing the model’s interpretability. In summary, Grad-CAM visualizations emphasize the key areas in the referee gesture recognition task, helping us to understand the model’s decision and focus, ultimately enhancing the model’s interpretability and visual analysis capabilities.

**Figure 13:**
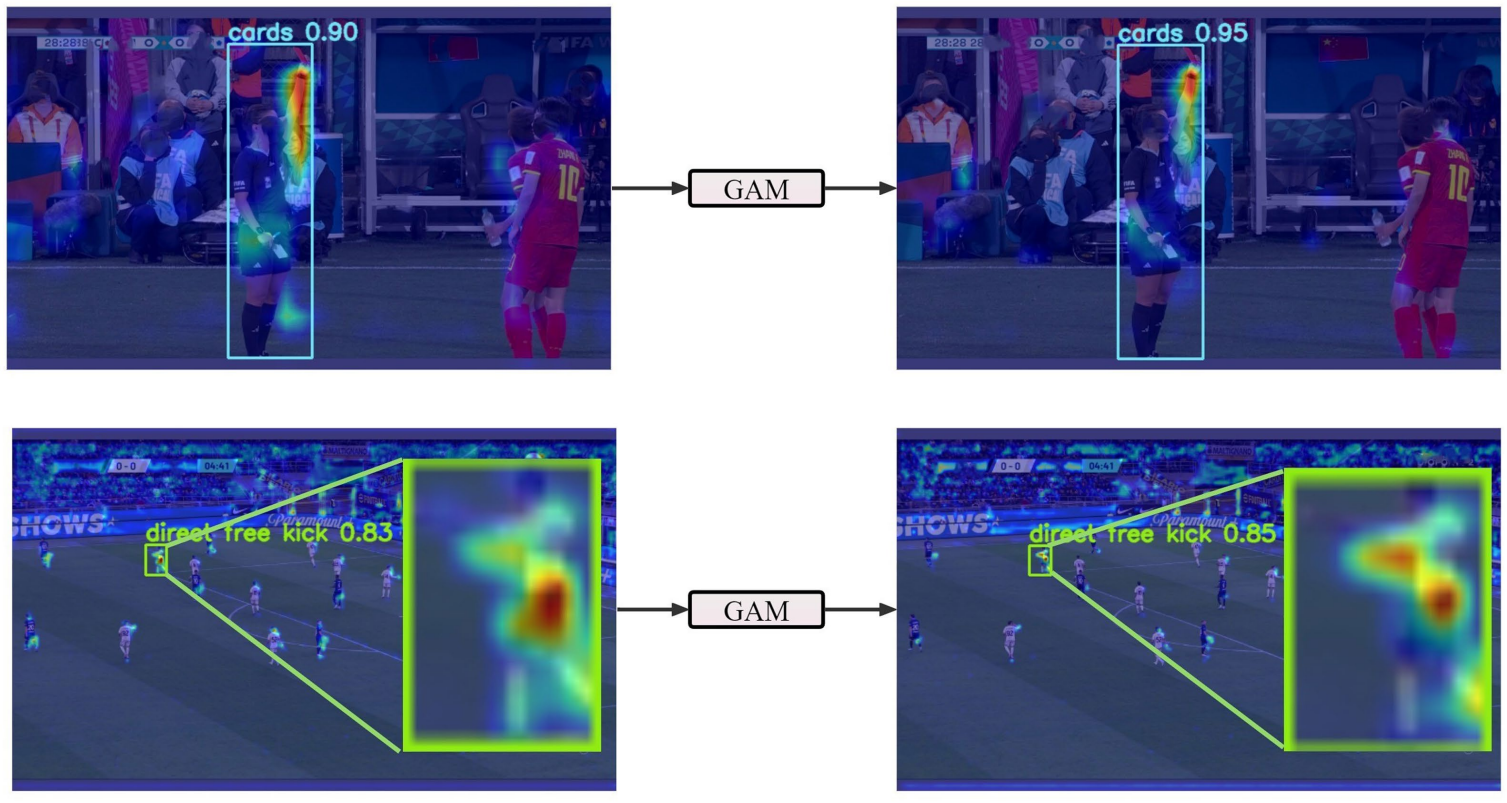
Grad-CAM visualization comparison for the input and output of GAM.

To better illustrate the role of the GAM module in football referee gesture recognition, we performed Grad-CAM visualizations on the input and output of the GAM module. As shown in Figure 13, we compared large and small targets separately. The images reveal that the attention points generated by the GAM module are centered around the position of the referee’s arm. Our model relies heavily on this area for recognizing referee gestures. Thanks to the GAM module, our model can focus more precisely on the regions relevant to referee gestures.

**Figure 14:**
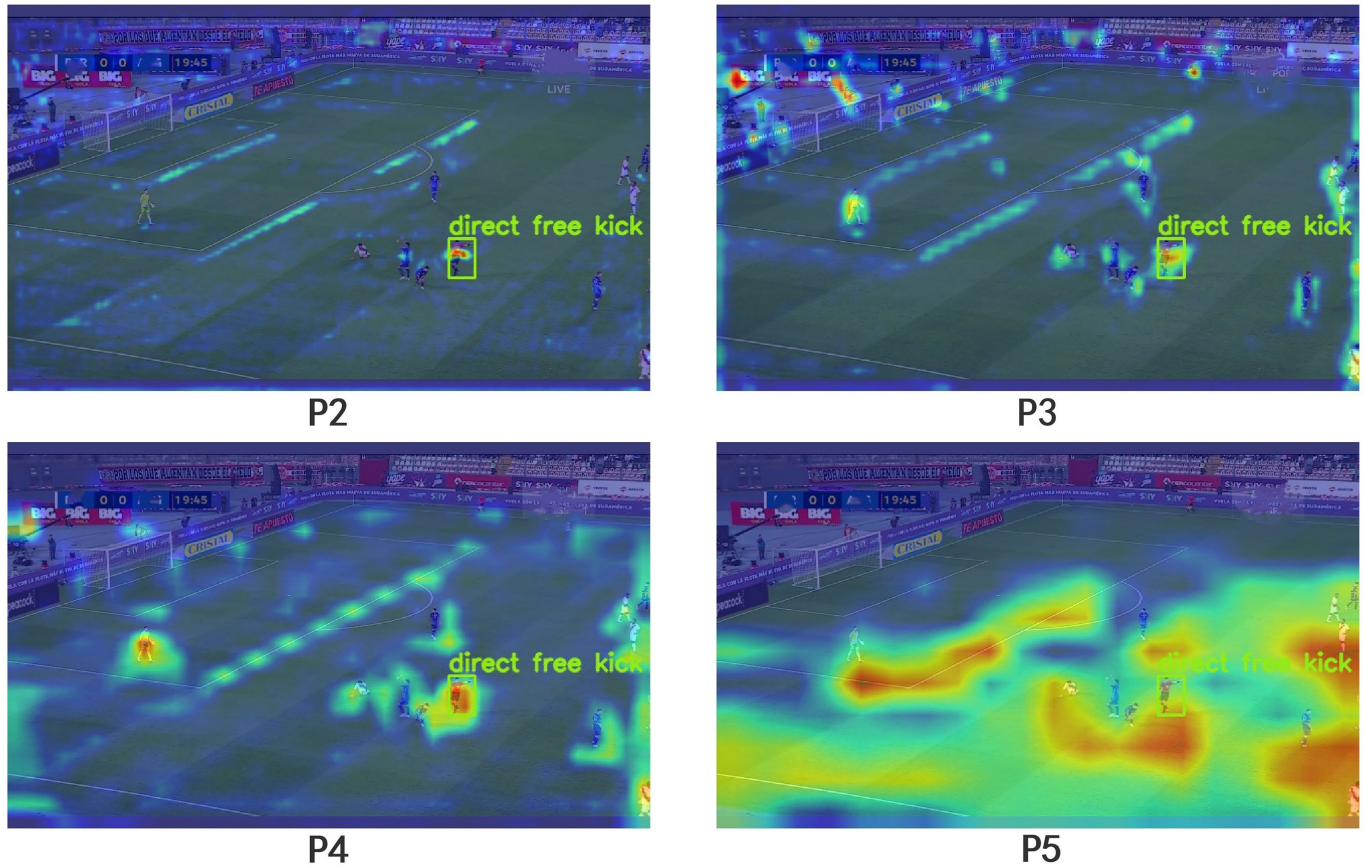
Comparison chart illustrating Grad-CAM for the detection performance of different detection heads.

To better demonstrate the efficacy of the P2 detection head in detecting small targets, we conducted a comparative analysis of Grad-CAM visualizations for different detection heads, as depicted in Figure 14. It is evident from the images that the P2 detection head is capable of focusing on minute details in the image, further substantiating its proficiency in small target detection.

#### 4.4.3 Comparison of the detection effect of long-distance camera images

**Figure 15:**
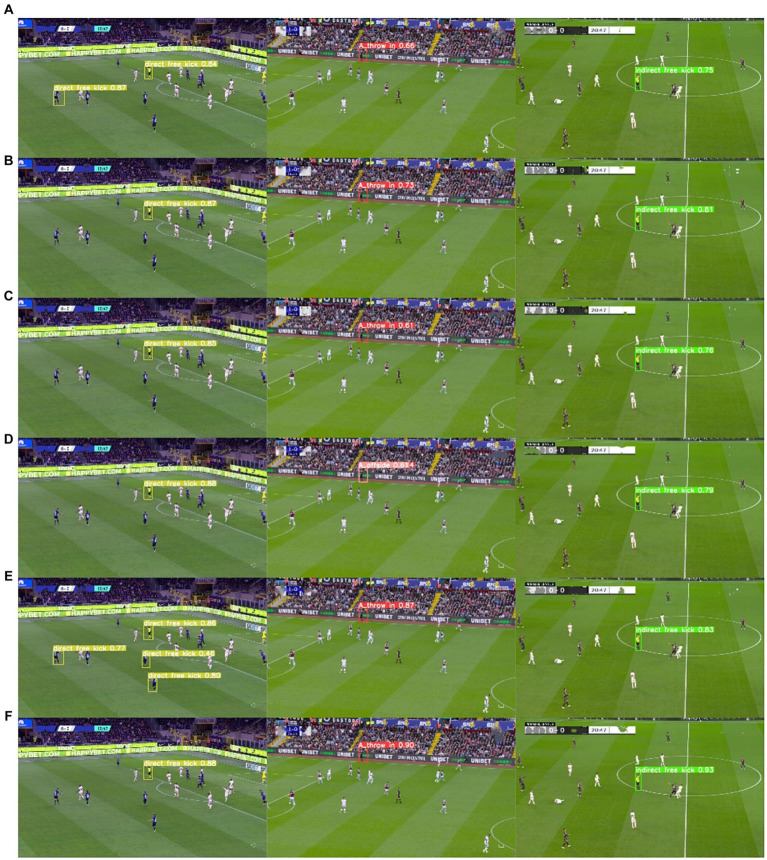
Comparison of small object detection performance across various algorithms. **(A)** YOLOv3tiny; **(B)** YOLOv5s; **(C)** YOLOv6s; **(D)** YOLOv7tiny; **(E)** YOLOv8s; **(F)** FRGR-YOLOv8s.

We also compared the performances of various algorithms in recognizing referees’ gestures captured with long-distance cameras. As shown in Figure 15, it is evident that FRGR-YOLOv8s outperforms the other algorithms, with a higher accuracy and lower false detection rates.

#### 4.4.4 Comparison of different IoU

This study conducted experiments by adding different IoU settings on the YOLOv8s + GAM + P2 model, as shown in Table 4. The table indicates that MDPIoU outperforms other IoU settings overall, providing further evidence of the effectiveness of MDPIoU in football referee gesture recognition.

**Table 4:** Comparison of different IoU.

Models	*P*	R	mAP@0.5	mAP@0.5:0.95
YOLOv8s + GAM + P2 + CIoU	88.2%	87.9%	90.2%	76.5%
YOLOv8s + GAM + P2 + DIoU	84.2%	88.4%	89.8%	74.4%
YOLOv8s + GAM + P2 + GIoU	88.9%	84.7%	90.2%	75.3%
YOLOv8s + GAM + P2 + SIoU	85.1%	87.6%	89.5%	74.1%
YOLOv8s + GAM + P2 + EIoU	86.3%	86.1%	88.2%	72.6%
YOLOv8s + GAM + P2 + MPDIoU (FRGR-YOLOv8s)	89.3%	88.9%	89.9%	77.3%

#### 4.4.5 Ablation experiments

To delve deeper into the impact of enhancing three distinct components on the network model’s performance, in this research, we carried out eight experiments. Each experiment involved the addition of different modules. Furthermore, comparative analysis was performed using evaluation indicators, and the results are presented in Table 5.

**Table 5:** Ablation experiments.

Models	*P*	R	mAP@0.5	mAP@0.5:0.95
YOLOv8s	87.9%	87.9%	88.8%	71.9%
YOLOv8s + GAM	89.7%	86.5%	89.6%	74.5%
YOLOv8s + P2	85.9%	89.8%	90.2%	75.7%
YOLOv8s + MPDIoU	88.9%	88.6%	89.0%	74.1%
YOLOv8s + GAM + P2	88.2%	87.9%	90.2%	76.5%
YOLOv8s + P2 + MPDIoU	84.7%	89.8%	90.7%	75.3%
YOLOv8s + GAM + MPDIoU	88.6%	87.0%	89.1%	72.9%
YOLOv8s + GAM + P2 + MPDIoU (FRGR-YOLOv8s)	89.3%	88.9%	89.9%	77.3%

Table 4 data reveals a significant improvement in the model’s P after integrating the GAM module. This improvement signifies that the GAM attention module prioritized the features related to the referee’s gestures during feature processing, thereby enhancing the model’s performance. The introduction of the P2 module enhanced the recognition of the referee’s gestures captured with long-distance cameras, minimizing the missed detections, thereby improving the recall rate. After incorporating MPDIoU, the precision rate significantly improved, suggesting that MPDIoU can effectively leverage the geometric properties of the detection frame in the referee’s gesture recognition, thereby enhancing the precision of target detection. In general, the addition of different modules has different effects on the model’s performance, but after comprehensive consideration, the FRGR-YOLOv8s model achieves the best overall detection result.

## 5 Discussion

In this paper, we conducted experiments using a self-constructed dataset derived from videos of multiple matches in the World Cup and other renowned football leagues. Each match showcased variations in lighting conditions, camera placements, and the attire of players and referees. The uniqueness of this dataset lies in its representation of the diversity across various match environments, including differences *in camera* layouts, variations in lighting conditions, and distinctions in referee attire colors. Training the model with such a dataset enhances its robustness, demonstrating strong performance across a wide range of complex scenarios.

By utilizing the YOLOv8s model as the foundation, we introduce the FRGR-YOLOv8s model to improve the precision and reliability of referee gesture recognition, making it versatile for diverse scenarios. Our research findings clearly indicate that the FRGR-YOLOv8s model excels when it is compared to the alternative models. In the field of object detection, various advanced models have emerged, including the two-stage object detection algorithm RCNN series, the one-stage object detection algorithm YOLO series, and SSD. While Faster R-CNN is a leading model in the RCNN series, its two-stage design restricts its applicability for real-time detection. Additionally, the SSD algorithm has a faster detection speed but demonstrates average performance in terms of detection accuracy. In relative terms, the YOLO series algorithms demonstrate superior performance in detection accuracy, as confirmed by comparative experiments. YOLOv8s, as part of the YOLO series, demonstrates optimal performance on the soccer referee gesture dataset. However, YOLOv8s also has its limitations in addressing these challenges, particularly when dealing with small targets and complex backgrounds within the dataset.

To address the challenge posed by complex backgrounds, we integrated the GAM on top of YOLOv8s. When comparing the Grad-CAM maps of the input and output of the GAM module, it was observed that the FRGR-YOLOv8s model more effectively focuses on the referee’s gesture in complex backgrounds. In tackling the problem of small targets, we introduced the P2 detection head in YOLOv8s. By comparing the Grad-CAM maps of different detection heads, it was noted that the P2 detection head more effectively emphasizes hand information in small targets. When comparing the FRGR-YOLOv8s model with the model lacking the P2 detection head, it was observed that FRGR-YOLOv8s performs better in detecting small targets. Additionally, we utilized MPDIoU in the YOLOv8s model to fully leverage the collective properties of detection boxes. Experimental results indicate that MPDIoU significantly enhances detection accuracy, particularly in comparative experiments with various IoU thresholds.

Compared to the YOLOv8s model, this model has shown a significant improvement in performance. The P increased by 1.4%, the R increased by 2.0%, the mAP@0.5 increased by 1.1%, and the mAP@0.5:0.95 increased by 5.4%. In general, the FRGR-YOLOv8s model outperforms the other models.

## 6 Conclusions and future work

The FRGR-YOLOv8s model introduced in this research surpasses the YOLOv8s model in the field of football referee gesture recognition. In the backbone network, we use GAM. The inclusion of the GAM module enhances the network’s capability to ex-tract features related to referees’ gestures, resulting in improved accuracy in detection. Furthermore, to enhance the detection performance of small objects, we introduced the P2 structure. MPDIoU was used instead of CIoU in the head network, which allows the better utilization of the geometric properties of the tag frame for football referees’ gestures. This helps in training the model and enhancing the accuracy of the predicted frame. Multiple experiments carried out on the referee gesture dataset validate that the FRGR-YOLOv8s model introduced in this paper showcases an exceptional performance. This model effectively bridges the gap in football referee gesture recognition.

While the FRGR-YOLOv8s model in our study has shown a significant improvement in performance for referee gesture detection compared to the YOLO series algorithms, it still has some limitations.

The FRGR-YOLOv8s model currently has technical limitations, as it can only recognize six common gestures made by soccer referees. In reality, the gestures of soccer referees encompass a wider range. In the future, we plan to enhance our dataset by integrating data from a broader range of match environments and including additional gesture categories. These enhancements aim to enhance the model’s flexibility and accuracy. Moreover, at this stage, the FRGR-YOLOv8s model has undergone specialized training and testing on a self-constructed dataset of soccer referee gestures. Its ability to accurately recognize gestures in other sports datasets remains unverified. Moving forward, we intend to collect gesture data from referees in various sports and evaluate the model’s capability to recognize gestures across diverse domains. Additionally, we intend to introduce a time-series module to enhance the functionality of the model, enabling it to recognize dynamic gestures.

## Data availability statement

The original contributions presented in the study are included in the article/supplementary material, further inquiries can be directed to the corresponding author.

## Author contributions

ZY: Conceptualization, Data curation, Formal analysis, Software, Validation, Writing – original draft, Writing – review & editing. YuS: Conceptualization, Data curation, Formal analysis, Funding acquisition, Validation, Writing – original draft, Writing – review & editing. YaS: Funding acquisition, Project administration, Resources, Writing – review & editing.
